# Congenital Aortic-Left Atrial Tunnel with Coarctation and Anomalous Left Coronary Artery from the Pulmonary Artery: A First-of-its-kind Case Report

**DOI:** 10.1007/s00246-022-02901-4

**Published:** 2022-04-11

**Authors:** Nikita Mittal, Aneela Reddy, Anita J. Moon-Grady, Elena K. Amin

**Affiliations:** 1 Department of Pediatrics, University of California, San Diego, CA USA; 2 Department of Pediatric Cardiology, University of California, San Francisco, CA USA

**Keywords:** Aortic-left atrial tunnel, Anomalous origin of the left coronary artery from the pulmonary artery, Coarctation of the aorta, Congenital heart disease, Fetal echocardiography, Angiography

## Abstract

Aortic-left atrial (Ao-LA) tunnel is an extremely rare vascular anomaly that involves an abnormal channel originating from the sinuses of the Valsalva and terminating in the left atrium. We present an unusual case of prenatally diagnosed Ao-LA tunnel with postnatal diagnosis of coarctation of the aorta and anomalous origin of the left coronary artery from the pulmonary artery (ALCAPA).

## Introduction

Congenital aortic-left atrial (Ao-LA) tunnel is an extremely rare vascular channel originating from the sinuses of the Valsalva and terminating in the left atrium. Few angiographic images of congenital Ao-LA tunnels have been published [1, 2]. Anomalous origin of the left coronary artery from the pulmonary artery (ALCAPA) is a more common isolated abnormality with a high mortality rate if left untreated in the first year of life [3]. Here we describe for the first time these two rare defects occurring together in the same patient with prenatal identification of the Ao-LA tunnel, postnatal identification of coarctation of the aorta and discovery of ALCAPA at the time of surgical repair.

## Case Presentation

**Fig. 1:**
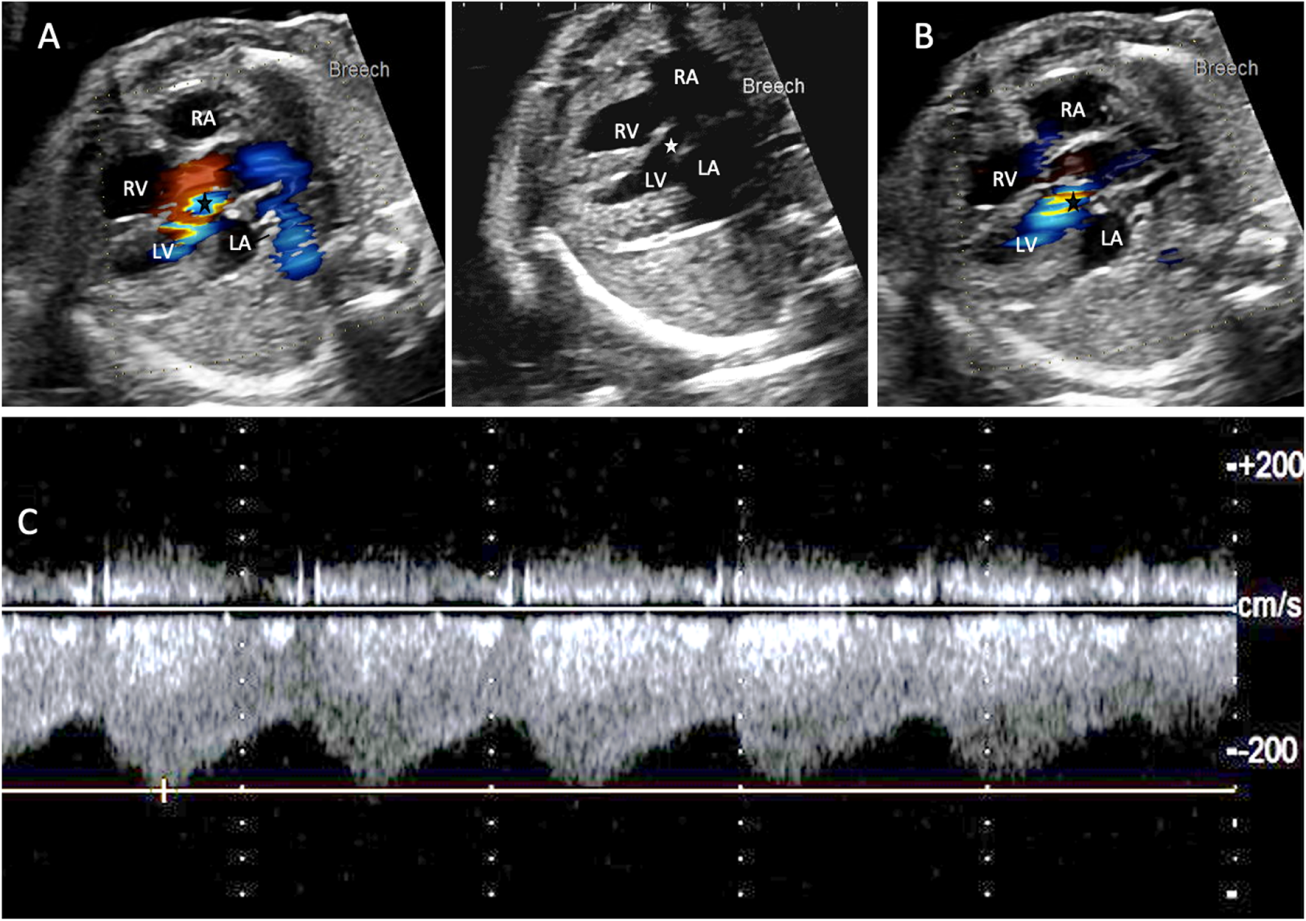
Fetal echocardiographic four-chamber view showing the Aortic-left atrial tunnel in both **a** systole and **b** diastole **c** Pulse wave doppler waveform obtained at the site of the aortic-left atrial tunnel (star) demonstrates a high velocity continuous waveform pattern diagnostic of this lesion. *RA* right atrium, *RV* right ventricle, *LV* left ventricle, *LA* left atrium

An Ao-LA connection was identified on fetal echocardiogram based on color and spectral Doppler patterns (Fig. 1) at 19 weeks gestation following referral for family history of bicuspid aortic valve. Serial fetal echocardiography showed no change in appearance with normal ventricular size and function, and no evidence of hydrops. Delivery occurred at 33 + 6/7 week gestation following preterm premature rupture of membranes. At birth, the infant was cyanotic and hypotonic improving with continuous positive airway pressure (CPAP) of 5 mmHg. Physical examination revealed a 2090 g, acyanotic, premature infant, with normal respiratory pattern and grade 3/6 medium frequency murmur at the left sternal border with radiation to the left axilla. Pulses were initially equal in intensity in all extremities. Postnatal echocardiogram revealed a small membranous ventricular septal defect (VSD), secundum atrial septal defect (ASD), bicuspid aortic valve, patent ductus arteriosus (PDA), normal biventricular systolic function, normal right coronary artery origin and left coronary artery (LCA) flow was not well seen. Continuous flow from the aorta to the left atrium was speculated to be either due to a direct Ao-LA connection, a coronary fistula or a ruptured sinus of Valsalva aneurysm. Tachypnea with pulmonary edema on chest x-ray was noted at one week of life with rising lactate level despite increased diuretics. The clinical picture was consistent with cardiogenic shock, disseminated intravascular coagulation and hypoxic liver shock. Repeat echocardiogram revealed discrete coarctation of the aorta and severe biventricular dysfunction. Clinical stabilization and dramatic improvement in lactate and hepatic function was achieved with endotracheal intubation, initiation of prostaglandin E1 infusion, vasoactive pressor infusions, sedation and transfusion of blood products.

**Fig. 2:**
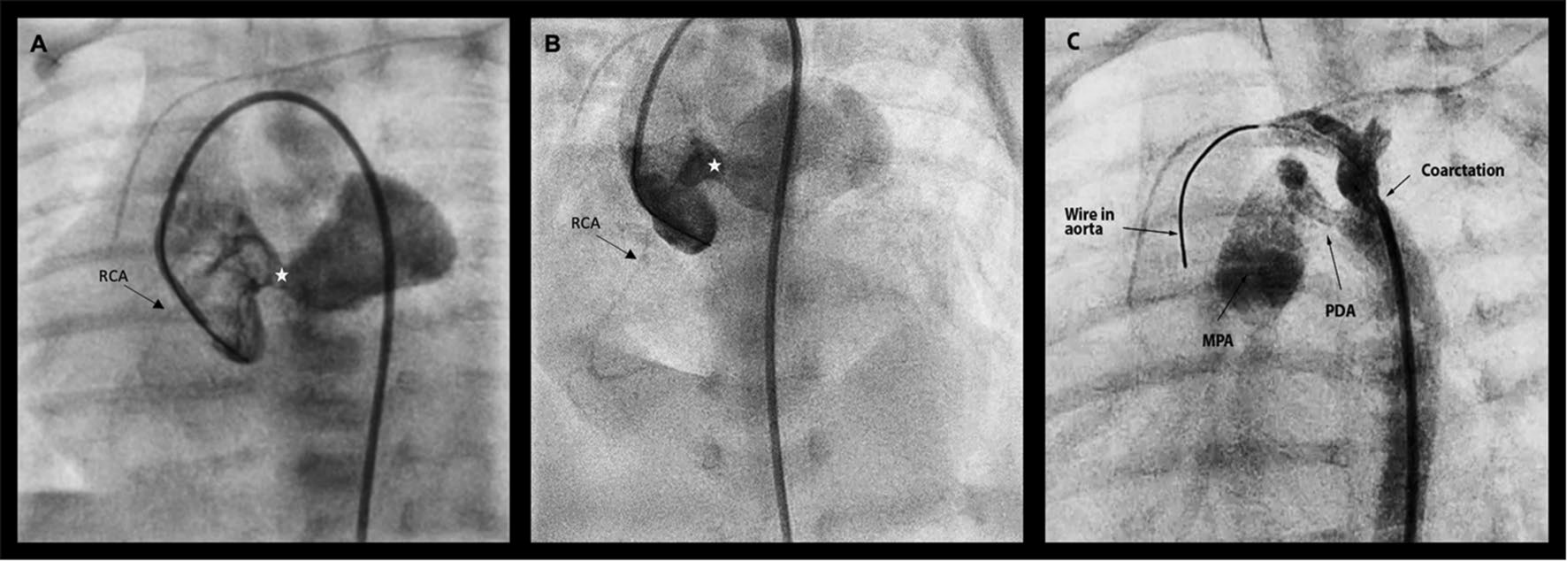
Ascending aorta angiogram with hand injection of contrast into the right aortic cusp in **a** straight AP projection and **b** right axial oblique 10, cranial 30 projection, via a 4-French non-tapered angle glide catheter advanced retrograde via the umbilical artery confirming diagnosis of aortic-left atrial tunnel (white star) with normal right coronary artery origin and course. **c** Hand injection of contrast in the transverse arch in LAO 17, cranial 7 projection delineating coarctation of the aorta (black arrow) with constricted but patent ductus arteriosus on prostaglandin E1 infusion. *RCA* right coronary artery, *PDA* patent ductus arteriosus

At two weeks of life and weight of 2.5 kg, cardiac catheterization was performed to delineate the type of Ao-LA connection and determine whether the LCA was involved as a fistulous connection prior to surgical intervention. To avoid femoral arterial injury, access was obtained via exchange of the existing umbilical arterial line for a 4-French non-tapered angle-glide catheter. Aortic root hand-injection angiography revealed a clear aortic-left atrial tunnel, normal right coronary artery origin and course without fistulous connections, and coarctation of the aorta with partially constricted PDA despite prostaglandin E1 infusion (Fig. 2). Selective injection was performed directly into left coronary cusp, however, the left coronary arterial system was not well delineated on any of the angiograms; this was presumed due to steal or compression from the AO-LA tunnel.

**Fig. 3:**
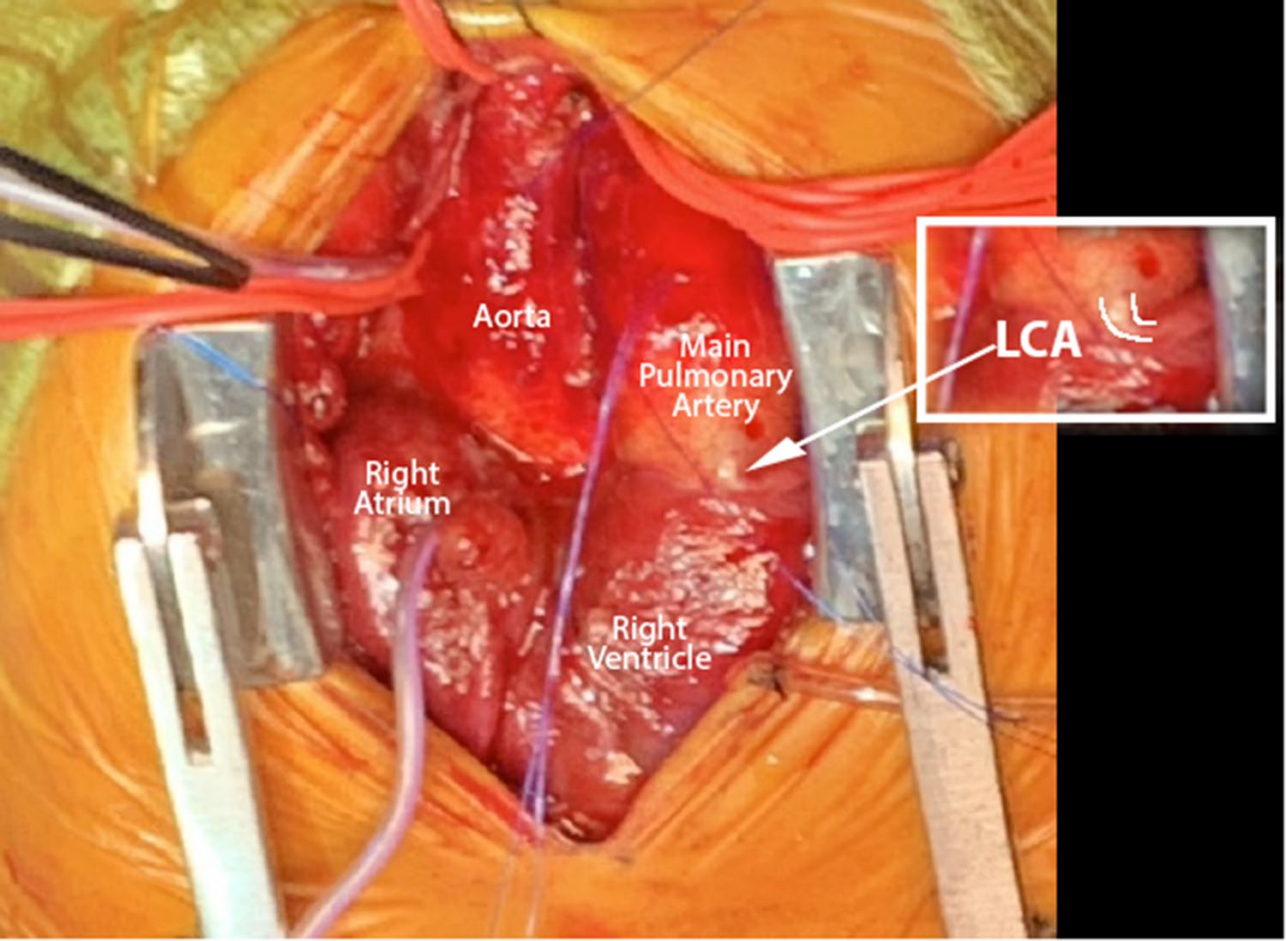
Intra-operative diagnosis of anomalous left coronary artery originating from the main pulmonary artery. *LCA* left coronary artery

Surgical repair was undertaken at 2.5 weeks of life and weight of 2.7 kg. Following median sternotomy and harvesting of the pericardial patch, the LCA was unexpectedly noted to arise anomalously from the anterior pulmonary artery (Fig. 3). The Ao-LA tunnel was located just superior to the sinotubular junction with no involvement of the coronary arteries. Suture closure of the tunnel was performed. The left subclavian artery (LSCA) was transected and anastomosed to the anomalous LCA. ASD and VSD closure were completed using autologous pericardium. The PDA was ligated and divided. Function by transesophageal echocardiogram was minimally depressed post long cardiopulmonary bypass time and the chest was left open for planned delayed sternal closure. Additional inotropic and vasoactive support was required in the immediate post-operative days with chest closure tolerated on post-operative day seven. Follow up echocardiograms have shown residual shunts and normal biventricular function. She was discharged to home without respiratory support on aspirin and diuretic therapy 39 days post-operatively at 42 weeks corrected gestational age. At 5 months of age, she is thriving at home, tolerating oral feeds without respiratory support or diuretic therapy.

## Discussion

Congenital tunnels between the aorta and atria are an extremely rare vascular anomaly with newborn clinical presentation ranging from clinically silent to cardiogenic shock [1, 2, 4, 5]. Aortic-left atrial tunnels have a much lower reported incidence than aortic-right atrial tunnels [5]. Most of these cases have been associated with concurrent cardiac anomalies such as atrial septal defects [2]. This is the first case to report a neonate with an aortic-left atrial tunnel with a concomitant ALCAPA, VSD, and coarctation of the aorta. Based on our patient’s course, this becomes especially significant as infants diagnosed with ALCAPA are at high risk for myocardial infarction, congestive failure, and early death at less than 1 year of life [3]. Although ALCAPA is most often an isolated anomaly it has been described in association with other cardiac defects, most commonly coarctation of the aorta [6].

Fetal echocardiography can play a vital role in early detection of these rare but potentially fatal anomalies allowing for advance delivery and postnatal care planning. Our patient was successfully diagnosed with an aortic to left atrial connection at 19 weeks gestation which allowed for careful prenatal monitoring as well as a coordinated delivery between the neonatology and cardiology teams. Fetal doppler patterns of the tunnel demonstrated both arterial and atrial pressure waveforms with high velocity continuous flow and therefore was diagnostic for this lesion (Fig. 1). The additional diagnosis of coarctation was likely unrecognized in fetal echocardiogram because the Ao-LA tunnel dilates the left heart and ascending aorta and therefore masks the right ventricle-left ventricle asymmetry and pulmonary artery to ascending aorta asymmetry which are associated with coarctation prenatally.

We speculate that the severe decompensation with partial ductal closure at one week of life was multifactorial, and that the rapid recovery of biventricular function after initiation of PGE1 was due not only to resolution of obstruction at the aortic isthmus but also increase in left coronary artery perfusion pressure once the main pulmonary artery pressure was restored to near systemic pressure with the initiation of PGE1. However, the decompensation noted at the time of coarctation diagnosis, and the rapid improvement thus biased the clinical investigations against a coronary issue and focused the diagnostic catheterization on the fistula and coarctation instead. The lack of filling of the left coronary on aortogram was attributed to competitive flow to and/or compression by the Ao-LA tunnel. Injection of contrast at the aortic ampulla of the PDA did not fully opacify the main pulmonary artery. Though ALCAPA is always considered when an LCA cannot be identified on angiography, the option to cross the PDA for angiography of the main pulmonary artery was not performed to avoid risk of PDA spasm that would disrupt systemic perfusion in the setting of coarctation. In retrospect, right heart catheterization with angiography of the main pulmonary artery would potentially have identified the ALCAPA prior to intra-operative identification.

Fetal echocardiography with doppler pattern analysis can identify an Ao-LA connections. At our institution, despite several repeat echocardiograms, the distinction between aortic-left atrial tunnel versus ruptured Sinus of Valsalva aneurysm was not discerned until the catheterization procedure. Ao-LA tunnels are reported to have a variety of concomitant congenital cardiac anomalies therefore a high suspicion for additional diagnoses should be maintained as these may have been masked on fetal or immediate postnatal echocardiography. This case report aims to highlight that despite both of these anomalies being extremely rare, absence of the LCA on aortic root injection despite a plausible alternative explanation should prompt specific evaluation for ALCAPA i.e. the probability of a concurrent Ao-LA tunnel and ALCAPA is higher than previously thought. Confirmation of coronary origins is prudent prior to attempting surgical repair of Ao-LA or aortic-right atrial tunnels as the coronary arteries may arise in the normal position, from an anomalous location or from the tunnel itself all of which may be difficult to determine solely by echocardiography [7]. Serial echocardiography and further evaluation to distinguish the type of aortic-atrial connection, coronary arterial anatomy and associated defects including cardiac catheterization with angiography should be performed as soon as possible to facilitate early diagnosis of associated anomalies and facilitate early surgical repair. In cases of isolated Ao-LA tunnel, transcatheter device closure is an alternative to surgical repair [1]. As the spatial resolution of 3-dimensional imaging modalities including CT angiography continues to improve this may be an increasingly available alternative to invasive cardiac catheterization for anatomic evaluation. Despite our patient’s prematurity, low birthweight and initial delay in identification of the complete spectrum of cardiac anomalies, she was able to undergo the necessary diagnostic studies and early successful complete surgical repair to avoid further clinical sequelae.

## Data Availability

Not applicable.
